# Integrated software for multi-dimensional analysis of motion using tracking, electrophysiology, and sensor signals

**DOI:** 10.3389/fbioe.2023.1250102

**Published:** 2023-11-22

**Authors:** Eis Annavini, Jean-Luc Boulland

**Affiliations:** ^1^ Division of Physiology, Department of Molecular Medicine, Institute of Basic Medical Sciences, University of Oslo, Oslo, Norway; ^2^ Department for Immunology, Clinic for Laboratory Medicine, Oslo University Hospital, Rikshospitalet, Oslo, Norway

**Keywords:** software, motion, integrated, electrophysiology, sensors, analysis, open source

## Abstract

Tracking followed by analysis of specific point-of-interest from conventional or high-speed video recordings have been widely used for decades in various scientific disciplines such as sport, physiotherapy, and behavioral science. Another method used to characterize movement in 3D involves the use of motion capture systems, which produce files containing a collection of 3D-coordinates and corresponding timestamps. When studying animal or human movement, combining motion tracking with other recording methods–like monitoring muscle activity or sensor signals–can yield valuable insights. However, manual analysis of data from these diverse sources can be time-consuming and prone to errors. To address this issue, this article introduces a new, free, and open-source software developed in MATLAB. This software can be used as-is, or developed further to meet specific requirements. Once the coordinates are imported, multiple tools can be used for data preprocessing, such as to correct mistakes that may have occurred during tracking because of software errors or suboptimal video quality. In addition, the software can import coordinates from multiple cameras and combine them into a unified data series. With these inputs, the software can automatically calculate kinematic parameters and descriptive statistics, generate 2D and 3D animations, and analyze gait cycles, enabling swift and accurate analysis of multidimensional motion data. Moreover, the software can import electrophysiology traces and sensor signals, which can be filtered, rectified, smoothed, and correlated with the kinematic data in various ways. Thanks to its user-friendly graphical user interface, the software is easy to navigate and can be used to analyze complex movements without any need for coding skills. This versatile tool is well-suited for a wide range of experimental contexts, making it a valuable resource for researchers across diverse scientific disciplines.

## Highlights

• Analyzes tracking data in 2D and 3D to evaluate motion
• Processes electrophysiology traces and sensor signals for an integrative movement analysis
• Includes multiple tools for preprocessing of data
• Features a user-friendly graphical interface, accessible without any coding knowledge
• Developed using MATLAB, but no MATLAB license is required for its use

## Introduction

Tracking the movements of animals, limb joints, single cells, and other subjects in space and time is an experimental method relevant to many scientific disciplines. In bioscience, it is so widely used that it is challenging to keep track of all the species that have been studied in this way. Some examples include oikopleura (Kreneisz and Glover, 2015), tadpole (Bacqué-Cazenave et al., 2018), zebrafish (Barreiros et al., 2021), rodents (Bachmann et al., 2013), chicken (Bari et al., 2021), cats (López Ruiz et al., 2017), pigs (Boakye et al., 2020) and non-human primates (Fitzsimmons et al., 2009). In neuroscience, biomedicine, and sports science, this method is typically used to compare kinematic quantities across different conditions. It is particularly relevant for the study of locomotion, motor systems, and related defects like spinal cord injury, stroke, traumatic brain injury, Parkinson’s disease, nerve injury, and others. Video tracking is, therefore, often used in comparative studies involving different groups (e.g., wild type, KO, injured, treatment), with high frame-rate video recordings of animals and humans performing specific motor tasks (e.g., running on a treadmill). In many cases, points-of-interest (POI) are tracked on a video recording to determine their position in space and time. Alternatively, a 3D motion capture system enables direct tracking of the POI. Further analysis of these data permits an objective and quantifiable evaluation of the movement, enabling reliable inter-group comparisons.

Numerous tracking programs are available, both free and open source as well as commercial licensed software. For tracking short sequences, one option is to use the popular Java-based program ImageJ, developed by NIH, which serves as a powerful imaging platform used by scientists in many research fields (Rasband, W. S., 1997; Schneider et al., 2012). The Manual Tracking plugin in ImageJ enables the recording of coordinates and the calculation of instant speed for multiple POI (Cordelières, 2006). However, tracking POI manually can be time-consuming, particularly with high frame-rate videos. Other plugins like TrackMate (Tinevez et al., 2017), MTrack2, MultiTracker, SpotTracker, MTrackJ (Meijering et al., 2012), Speckle TrackerJ (Smith et al., 2011), and ToAST (Hegge et al., 2009) aim to provide automated, semi-automated, and manual tracking of single-particles/objects. Though these plugins can be used for motion tracking of moving animals, they may be more appropriate for time-lapse microscopy imaging. Other free programs offer automated tracking, including, Kinovea (Puig-Diví et al., 2019), OptiMouse (Ben-Shaul, 2017), Toxtrac (Rodriguez et al., 2018), Tracker (Brown and Cox, 2009; Nakamichi and Asahara, 2022), MouseActivity (Zhang et al., 2020), Traktor (Sridhar et al., 2019), idTracker (Pérez-Escudero et al., 2014), Ctrax (Branson et al., 2009), BioTracker (Mönck et al., 2018), UMATracker (Yamanaka and Takeuchi, 2018), BEMOVI (Pennekamp et al., 2015), FIMtrack (Risse et al., 2017), ezTrack (Pennington et al., 2019), Whisker-Tracker (Azarfar et al., 2018), YOLOv2 (Barreiros et al., 2021), OpenPose (Cao et al., 2021), PoseNET (Erfianto et al., 2023), AlfaPose (Mehdizadeh et al., 2021; Fang et al., 2023), DeepPoseKit (Graving et al., 2019), idtracker.ai (Romero-Ferrero et al., 2019), B-KinD (Sun et al., 2022), TRex (Walter and Couzin, 2021), DeepLabCut (Nath et al., 2019), B-SOiD (Hsu and Yttri, 2021), LEAP and SLEAP (Pereira et al., 2019; 2022), and DeepLabStream (Schweihoff et al., 2021).

In some cases, their tracking accuracy may depend on image quality, and on the use of high-contrast markers such as retroreflective beads or tape, or permanent or temporary tattoos; These can facilitate tracking and improve accuracy. The most recent programs use machine learning and deep neural networks to enable markerless tracking of single and multiple animals. The accuracy of these software packages has been validated by various studies, that have used different subjects and different programs (Mehdizadeh et al., 2021; Stenum et al., 2021; Johnson et al., 2022; Lonini et al., 2022; Washabaugh et al., 2022; John et al., 2023; Lee et al., 2023). In addition, other interesting initiatives have resulted in gathering various open-source resources, including analysis tools and downloadable datasets, under the same repositories. Examples include OpenBehaviour (https://edspace.american.edu/openbehavior/) and a GitHub repository maintained by Dr. Luca Modenese (https://github.com/modenaxe/awesome-biomechanics). Apart from open-source software, there are also multiple systems available for purchase, with some requiring specific hardware—as determined by the seller—while others do not have such requirements. Notable examples are WINanalyse (https://winanalyze.com/), ProAnalyst from Xcitex (http://www.xcitex.com/), visual 3D from C-Motion (https://c-motion.com/), Vicon (https://www.vicon.com/), Delsys (https://delsys.com/), The Motion Monitor (https://innsport.com/), and Qualisys (https://www.qualisys.com/). These systems can propose advanced motion analysis and modeling features, but they come at a cost that may not be affordable for everyone.

Preprocessing and analysis of data from 2D–3D coordinates to obtain multiple kinematic quantities, various diagrams, descriptive statistics, animations, and gait parameters can be a tedious and time-consuming process. In biomedical science, it can also be valuable to combine 2D–3D coordinates with electrophysiology and/or sensor signal recordings to analyze the function of specific muscles or determine the force applied in a defined phase of the movement. The Integration of these analyses provides an overview of the biomechanical and physiological aspects of movement. The use of spreadsheet programs for these tasks requires a great number of actions and is potentially prone to mistakes. Therefore, to make this work easier and to standardize the analysis, we created a MATLAB program called MotionAnalyser. Although MATLAB is an important research tool, a lack of coding experience may present a barrier for a significant number of users. Which is why we created a graphical user interface (GUI) aimed at providing a simple and user-friendly experience for comprehensive, accurate, fast, and reproducible analysis. The GUI is organized with task-dedicated tabs. The *Coordinates* tab enables preprocessing of 2D and 3D coordinates, which can then be animated in the *Animation* tab. Further calculations and plotting can be automated from the *Kinematic* tab, and descriptive statistics can be obtained from the *Stat* tab. In addition, other signals—such as electromyograms and force plate data—can be preprocessed and analyzed in the *E-Phys* and *Sensor* tabs. All this data can then be correlated using different mathematical models in the *Correlation* tab. The *Stride & Gait* tab enables the identification of specific limb positions relevant for the calculation of different gait cycle parameters. All data generated can be exported in multiple file formats, including a. mat file that can be reloaded to MotionAnalyser for subsequent analysis.

## Materials and methods

### Software requirements

MotionAnalyser runs on MATLAB R2023a or later. Source code and application installers are available at https://github.com/BoullandLab/MotionAnalyser.

### Data loading

MotionAnalyser can handle any number of datasets, subject only to the maximum memory constraints of the operating system and of MATLAB. For example, a user interested in locomotion can import coordinates for the left and right legs and arms, and work with them either independently or together. Currently, the program supports loading data from spreadsheets (.xls/.xlsx), text files (.csv/.txt), and DeepLabCut (Nath et al., 2019) output files. In all other cases, data must contain appropriate headers and be organized column-wise, with timestamps being in the first column and spatial coordinates after (x, y), with the inclusion of the optional “z”coordinate for 3D. Different POI can either be imported from separate files or concatenated in a single file. All other data (EMG, sensors, etc.) follow the same schema. Data from the headers (tracked point, muscle, sensor, etc.) is used to label all tables and plots. Example data files can be downloaded together with the program (https://github.com/BoullandLab/MotionAnalyser).

### Data preprocessing

Coordinates obtained from video tracking or motion capture are susceptible to errors, including outliers. MotionAnalyser facilitates the identification, removal, and replacement of these outliers by interpolation, nearest value, mean window, previous value, etc. (Supplementary Figure S1A). It is also possible to apply different filters, such as the root mean square, moving means, etc., to smoothen out the trajectory.

Furthermore, traces acquired from multiple cameras capturing the same object may exhibit misalignment (Supplementary Figure S1B). MotionAnalyser provides a range of tools to correct this problem. In this process, it can be an advantage to work with separated datasets. As MotionAnalyser can import different elements separately, it enables the plotting, animating and preprocessing of each element independently, and without a segment joining them (Supplementary Figure S1C). The options mentioned in the rest of this paragraph can, therefore, be applied to individual elements. For instance, to compensate for mirror images acquired by cameras in opposite positions, the x-coordinates can be inverted (Supplementary Figure S1D). Other potential misalignments of opposite cameras can also be rectified by shifting coordinates (Supplementary Figure S1E). An auto-align option can also be used with a particular reference point indicated by the user. Perspective distortions might occur in case of camera misalignment or if a subject moves closer to one of the cameras, resulting in elements of different sizes, as exaggerated in the Supplementary Figure S1F. To restore proportions, a scaling factor can be applied to the set of coordinates sought. It is also possible to focus the analysis on a specific part of the movement by selecting the first and last desired positions before trimming the dataset (Supplementary Figure S1G). Other options, such as rotation, deletion of values, renaming the different POI, and others are also available. All changes are automatically saved and used in all downstream analyses without affecting the raw files. However, when using the downsampling option, for instance, to mitigate issues with overloaded graphs (Supplementary Figure S1H), it solely impacts the display and does not alter the data used for other calculations.

### Stitch

To combine two sets of coordinates recorded by cameras on the same side of a subject, MotionAnalyser enables their stitching. First, the user imports the initial set of coordinates and then imports the stitch set, which is plotted alongside the initial set (Supplementary Figures S2A, B). The user can adjust the position of the stitch coordinates and rotate them to correct for a camera misalignment. The stitching option enables the user to choose the transition point in the stitch sequence (Supplementary Figures S2C, D). Note that MotionAnalyser does not provide a function for camera calibration to correct distortions at the overlap between two cameras. It is necessary to correct this before importing the data into MotionAnalyser.

### Animations

Users can animate the different elements independently or together and control various display parameters (Supplementary Video S1). For example, users can adjust the animation speed, the perspective, change the line thickness, and add or remove a marker of the desired color, so as to indicate the positions of different POI. It is also possible to normalize the movement on different planes by defining a POI as a reference. When using 2D data, an arbitrary value is assigned to the third coordinate of each POI (by default, 0). These values that can be adjusted by the user, are only used for visualization purposes, and are never used in the calculation of kinematic parameters.

### Calculation of kinematic quantities

Before calculating the kinematic quantities, the user has the option to choose to set the expression of speed and acceleration as absolute values to avoid negative speeds and accelerations. The default expression of angles can be changed from degrees (default) to radians. The mathematical equations used for the calculation of the kinematic quantities are shown in the Supplementary Table S1. MotionAnalyser determines extrema values, i.e., minimum, maximum, and it calculates and plots descriptive statistics, i.e., mean, median, and mode values. These values, however, are not displayed on the *Kinematic* tab but can be queried and saved from the *Stats* tab. If there are two or more POI, MotionAnalyser can create a stick diagram of the movement. This diagram includes a segment between the POI for each time value. The stick diagram can also be normalized on the *x*-axis, on the *y*-axis, or both using a defined reference point that can be changed by the user. This enables visualization of the movement with a fixed anchoring point. This feature eliminates fluctuations, which can be useful for suppressing movements that are unrelated to the specific movement of interest, e.g., an animal swimming. Note that this normalization only affects the display, but it does not modify the calculation of kinematic variables. The same option is also available for the *Animation* and the *Stride & Gait* tabs.

### Electrophysiology and sensor signal analysis

The electrophysiology and sensor signals are treated similarly, although they are displayed on different tabs to allow easy switching between them. These signals can be processed in various ways, including trimming, adjusting temporal resolution, resampling, interpolating, filtering, rectifying, and creating a signal envelope (Supplementary Table S1). Adjusting the temporal resolution should not be confused with resampling or interpolation; it simply involves changing the time array to a user-defined value. This can be particularly useful in cases where time values are missing in the raw data, but the sampling rate is known, which can occur with certain sensors. MotionAnalyser can also perform spectral analysis of the signal using Fast Fourier Transform (FFT) and can plot the power spectrum and spectrogram of the signal (Supplementary Figures S3A, B). Digital filtering is calculated using the Butterworth digital filter function with an adjustable filter order. MotionAnalyser also provides several rectification options, including mean rectification, absolute value rectification, square root rectification, and half-wave rectification (Supplementary Figures S3C, D; Supplementary Table S1). To generate a signal envelope, users can use a low-pass Butterworth filter or the Hilbert transform, both programmed with existing MATLAB functions. Alternatively, users can also use a moving average or root-mean-square of the signal with an adjustable window (Supplementary Figure S3E). In addition, users have the option to calculate the area under the curve of the signal. If required, it is always possible to revert to the original signal, allowing for experimentation with various signal processing methods.

### Correlations

This tab enables the user to correlate kinematic quantities, either within elements or between different elements. This feature can be useful for plotting the cyclogram for two joint angles. In addition, the user can correlate the electrophysiology and sensor data between each other or with the kinematic quantities. In some cases, resampling or interpolation and trimming are necessary to ensure that the sampling rates and sequence duration are consistent. However, the user maintains control throughout this process, as a dialogue box provides guidance. MotionAnalyser also calculates the autocorrelation function of any of the imported signals to determine its potential rhythmicity. The cross-correlation function correlates two different signals to determine the degree of similarity and coordination between them. The phase lag and the amplitude ratio, which are calculated using MATLAB’s algorithm for FFT, are also calculated. The amplitude ratio is determined by calculating the ratio of the peak amplitude for each signal, i.e., the maximal value of the FFT for each signal. The phase lag is obtained by computing each signal’s phase at peak frequency using MATLAB’s angle function, and then taking their difference. Furthermore, MotionAnalyser offers additional methods for comparing two signals of the same duration and sampling rate: Euclidean distance, cosine similarity, Pearson’s, Spearman’s, and Kendall’s correlation coefficients. In cases where resampling or interpolating the signals is not feasible or cannot be accomplished satisfactorily, MotionAnalyser provides an alternative option for assessing the correlation of two signals using Dynamic Time Warping. This method requires the user to adjust the size of a moving window to manage the computational load in accordance with the available computing power.

### Locomotion analysis

The *Stride & Gait* tab is specifically designed for carrying out the analysis of locomotion and represents the most interactive area of the software. Users can navigate through different limb positions from the control panel to identify the specific positions that characterize the gait cycle. A classic decomposition of the gait cycle is used by MotionAnalyser (Figure 3A), which includes a swing phase between the toe-off and heel-strike positions and a stance phase between the heel-strike and the next toe-off position (Dale, 2012). The program calculates several parameters, including the stance and swing phase durations, stride length, and duration, which are particularly useful when analyzing a defective locomotion. MotionAnalyser also plots a gait diagram. Two additional limb positions can be recorded if desired; the heel-off to calculate the heel-down duration as part of the stance phase, and the maximum toe-lift to determine the toe-lift speed and duration. To obtain reliable data, it is essential to select the heel-strike as the starting point of the gait cycle. The software can analyze an unlimited number of cycles, with each position being displayed in a distinct color that can be customized. In addition, the coordinates and corresponding time values are presented in a table for easy reference. As for the *Animation* and the *Kinematic* tabs, the movement can be normalized to a chosen point of interest on the *x*-axis, the *y*-axis, or both. The stick diagram used to identify the relevant limb position can be zoomed in and out to accommodate many gait cycles. The pan tool that appears when dragging the mouse over a background area of the diagram can be used to re-center an area of interest in the diagram.

### Exporting analyzed data

After completing the analysis, users can export all analyzed data to multiple xls/xlsx/txt/csv files. It is also possible to save the data as a mat file, which enables users to further analyze the data in the MATLAB workspace, or reload the data in MotionAnalyser for a second analysis. Another advantage of the mat format is that it saves all data from position, tracking, electrophysiology, sensors, and gait into a single file. It is also possible to save all currently plotted figures. However, this function will not save previously plotted figures that were subsequently replaced. For saving sequential plots, users can save them manually one after the other, using the save option that appears on the plot when placing the mouse cursor over an empty area at the time of the plotting. Plots can be saved as image or vector graphic files. Animated models can be exported as individual AVI or animated GIF files.

### Note on units of measurement

MotionAnalyser is agnostic to units of measurement. Therefore, derived quantities follow from the units of measurement of the raw data. For example, if the input file has coordinates given in mm and time in seconds, the output speed will be in mm/sec. Nevertheless, it is always possible to adjust their order of magnitude using the scaling function (e.g., mm to cm).

### Software benchmarking

To evaluate the computing speed of MotionAnalyser, we performed MATLAB benchmark tests on a Windows 10 PC that featured an Intel Core i5-6500 running at 3.2 GHz, 16 GB of RAM, and an Intel HD Graphics 530 GPU. The tests involved five input files containing 13 columns, including time, and x- and y-coordinates for six POIs, for a total of 1,300, 13,000, 130,000, 1.3 million, and 13 million values.

### Software accuracy test using mathematical functions

All functions of MotionAnalyser were rigorously tested using signals generated from mathematical functions. These known mathematical properties provide a solid foundation to ensure that the outcomes of the calculations performed by MotionAnalyser are correct. For testing basic kinematic calculations, we generated two sets of data, where the x coordinate increases linearly with time, while the y coordinate increases quadratically. These respectively lead to a constant and linearly increasing speed, and a null and constant acceleration. In addition, we introduced a single outlier in one of the y-arrays. To test the accuracy of the angle calculation, we created a series of segments with known angles between them at 0, 90, and 135°. To test the gait analysis code, we created a dummy sequence of known dimensions for left and right legs. To test the treatment of electrophysiology and the sensor signal data, we created sine waves of known frequencies and amplitudes and applied successive filters while examining their effect on the FFT. We also tested the code that calculates the different correlation functions, amplitude ratio, and phase lag using two sine waves of a known phase shift of π radians and a known amplitude ratio of 2.

### Software accuracy test using real data

To further test the accuracy of the calculation, we used a real dataset obtained from one of our previous studies involving a mouse swimming experiment (Züchner et al., 2018). We compared the outcome from MotionAnalyser with alternative calculation methods like ImageJ, spreadsheets, and manual measurements.

### Example of use

We provide six examples of use of MotionAnalyser with real data obtained from various types of movements recorded in mice and humans. We generated all the mouse data during previous studies. For the human examples, we created the walking pattern data (Example 4) and the pendulum data (Example 5) to illustrate the potential use of MotionAnalyser. In the case of Example 6, we obtained the 3D motion capture and EMG datasets from a repository.

Example 1: Freely swimming 9-day old neonatal mice in a 210 × 165 mm pool for 20 s were filmed at 25 Hz. The movement was tracked with ImageJ and the Manual tracking plug in. This dataset comes from a previously published study (Di Bonito et al., 2015).

Example 2: Vestibular spinal test was performed on a 5-day-old neonatal mouse. This test entails placing the mouse in a device that rotates 90° from a straight position along the sagittal axis of the mouse. This simulation of falling, triggers the extension of the hindlimb on the side of the fall (Di Bonito et al., 2015). The video recording was at 200 Hz and tracking was performed as in Example 1.

Example 3: Adult mouse linear swimming, filmed from the side. The right limb joints were tracked with ImageJ as described earlier (Züchner et al., 2018). Recording and tracking parameters were similar to those described in Example 2.

Example 4: Video tracking from a human subject walking on an horizontal plane. Videos were recorded using two GoPro 7 Black cameras positioned on the left and right sides, on an axis roughly perpendicular to the line of progression. The cameras were connected to a remote control that synchronized them. The video were acquired at 60 Hz because the movements involved were slow enough. To mark the different joints on the legs, round markers of 3.5 cm diameter were cut out from a pink sheet of paper and attached with double-sided tape. Tracking was done using Kinovea.

Example 5: The leg pendulum test is sometimes used in clinics to determine the degree of spasticity of a patient (Biering-Sørensen et al., 2006; Aleksić and Popović, 2022). This is typically done with the patient sitting or lying down and the leg hanging over the edge of the couch, and then the examiner extends the leg horizontally. When released, the leg descends and rotates around the knee axis in a pendulum-like movement. This test was performed in a healthy subject combining the video camera recording at 120 Hz with a goniometer recording at 100 Hz (Moti, Denmark).

Example 6: 3D motion capture and EMG recording of a human arm during a flexion-extension. This movement involves bending the arm (flexion) and then straightening it back to the starting position (extension). The data were a subset of a larger dataset (Averta et al., 2020), case 01_10_2 in the H2 group.

## Results

**FIGURE 1:**
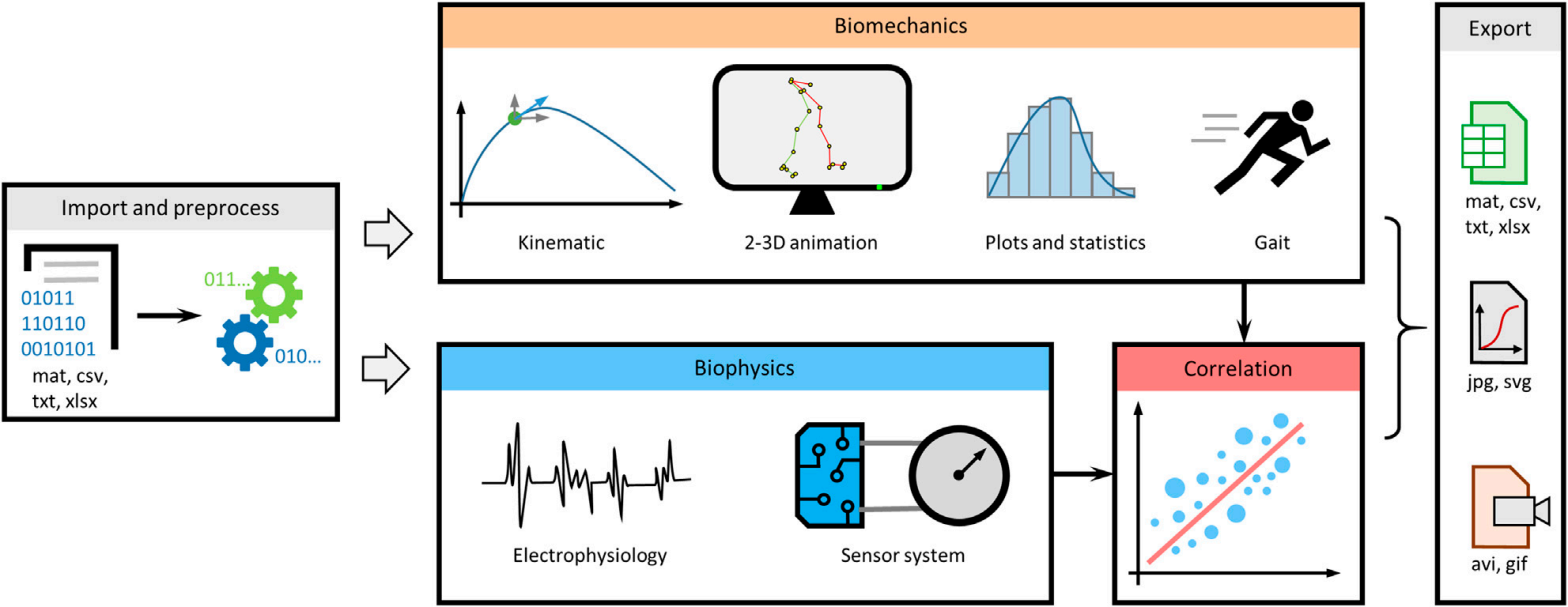
Overview diagram summarizing the different analyses that can be performed by MotionAnalyser. The program encompasses four primary analysis domains, namely, kinematics, electrophysiology, sensor signals, and stride and gait analysis. In addition, it enables correlation of results with and across each domain, allowing for comprehensive analysis.

MotionAnalyser is built around task-oriented tabs, enabling multimodal analysis of movement based on video tracking or motion capture, electrophysiology traces, and signals from sensor systems, such as pressure sensors, goniometers, and accelerometers (Figure 1). It includes multiple features to preprocess data, analyze kinematic parameters, create animations, and calculate descriptive statistics. It also enables analysis of electrophysiology and sensor signals. Various correlation functions can be used to better understand different aspects of the movement. In addition, MotionAnalyser enables gait analysis. Once the analysis is completed, the data can be exported to various formats, including spreadsheets, vector- or image-based graphics and animations.

Benchmarking MotionAnalyser on a mid-range computer showed, unsurprisingly, that some functions are slower when dealing with a high number of values, while others are less affected (Supplementary Figure S4). Loading data takes 83 s for 13 million data points (Supplementary Figure S4A). Plotting stick diagrams is the most resource-intensive operation, beginning to slow-down at 130k values before running out of memory at 13 M values (Supplementary Figure S4B). When saving data, mat files provide the highest performance; in contrast, saving csv files or spreadsheets is significantly slower (50s and 200s respectively), and the system runs out of memory with datasets of 1,3 and 13 M values. Other functions are affected differently by dataset size (Supplementary Figure S4D).

The program was further tested using synthetic data with predetermined properties. When the x-component of the array increased linearly, the program calculated a constant x-speed and x-acceleration was null, as expected (Supplementary Figures S5A, B). When the y-coordinates exhibited a parabolic increase, the program calculated a linearly increasing y-speed and a constant y-acceleration, as expected as well. Introducing a single-point perturbation in the y-array resulted in corresponding perturbations in the y-speed and y-acceleration, which are correct, as confirmed by manual calculation. Note that the two otherwise identical curves have been slightly shifted for visualization purposes. When tested on a synthetic dataset that generates known angles, MotionAnalyser correctly computed the corresponding angles (Supplementary Figure S5C). Similarly, by creating artificial arrays for the position of the toe, heel, and knee, a gait cycle was reproduced, yielding a stride length of 7 and a maximum toe-lift of 0.5 (arbitrary units), consistent with MotionAnalyser’s calculations. The gait diagram also correctly displayed stance and swing durations of 4 and 2 s respectively (Supplementary Figure S5E). To further assess the various components of the software responsible for calculating kinematic parameters, we used arrays derived from actual tracking data of a mouse hindlimb during linear swimming. The results showed that the calculated values were identical to those obtained through other means, such as ImageJ, spreadsheets, and manual calculations (Supplementary Figures S5F, G). The calculation of gait parameters from the tracking coordinates of a human subject were also identical to the manual measurements performed in Kinovea.

To evaluate the functions available for analyzing electrophysiology traces or sensor signals, we generated a signal from three sine waves at 25, 30, and 35 Hz. By applying two notch filters at 30 and 35 Hz, the original signal was correctly filtered, as evidenced by the absence of the corresponding frequencies in the power spectrum (Supplementary Figures S6A, B). The auto- and cross-correlation functions were tested with sine waves with known properties (Supplementary Figure S6C). The autocorrelation of each function showed a decaying cosine wave shape with the same period, as expected. As these two curves overlap, we slightly shifted one of them for visualization purposes (Supplementary Figure S6D). The cross-correlation correctly detected that the two functions are in antiphase (negative correlation), consistent with the calculation of a phase shift of 180° at lag 0. MotionAnalyser accurately calculated an amplitude ratio of 2, in agreement with the anticipated outcome.

**FIGURE 2:**
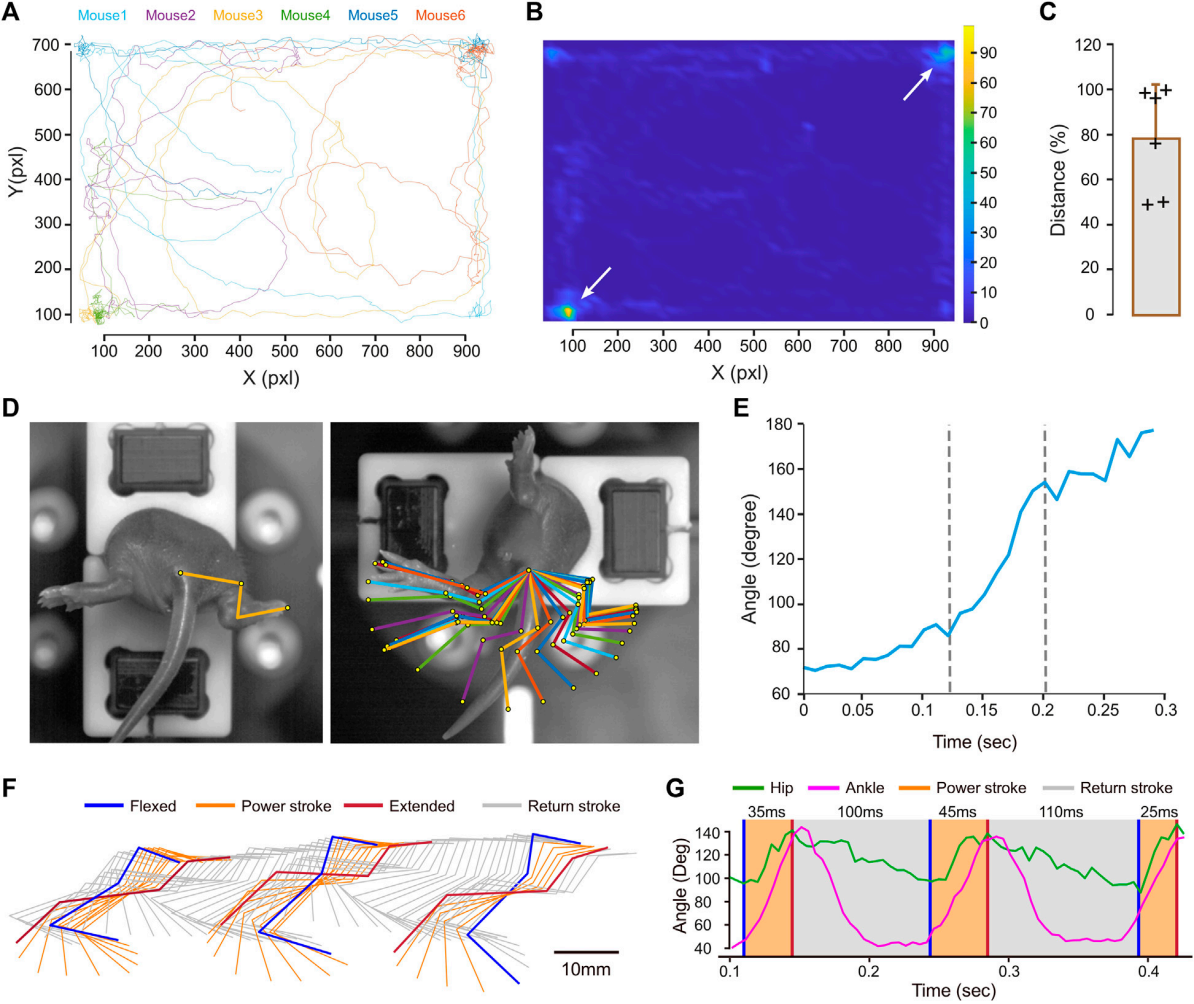
Examples illustrating the use of MotionAnalyser for analyzing animal movements. **(A)** Trajectory for 6 neonatal mice during free swimming. **(B)** Heatmap highlighting the hotspots for the trajectories depicted in **(A)**. **(C)** Total displacement of each mouse. The error bar represents the standard deviation. **(D)** Images showing the initial and final positions of a neonatal mouse undergoing the vestibulospinal test. These are overlaid with a stick diagram depicting the sequential positions of the hindlimb during the movement. **(E)** Temporal angular variation of the paw-ankle-base tail angle during the vestibulospinal test. **(F)** Stick diagram illustrating the sequential positions of the hindlimb during a linear swim, recorded from the side of an adult mouse. The power stroke is depicted in orange, while the return stroke is represented in gray. **(G)** Temporal angular variation of the hip (green) and ankle (magenta) during linear swimming.

MotionAnalyser was next used on experimental data. All the plots presented in this context were generated by MotionAnalyser. The first example involves tracking of six neonatal mice (9-day-old) during a free-swimming test conducted in a confined pool. Although the trajectories of the mice were diverse, they exhibited two distinct hotspots located at opposite corners, as depicted by the heat map (Figures 2A, B). The limited observation time of 20 s and the relatively small sample size (*n* = 6) may have hindered the detection of other potential hotspots. The distance swam by each mouse displayed variability. Despite this, a potential trend toward short- and long-distance swimmers was observed, although a larger sample size would be necessary to validate this observation (Figure 2C). MotionAnalyser also enables the extraction of additional kinematic parameters like speed and acceleration, thereby facilitating further characterization of each mouse and the potential grouping of individuals into distinct categories.

The second example illustrates the assessment of the vestibulospinal reflex in a 5-day-old mouse. The rapid rotation induced by the device holding the mouse triggers hindlimb extension (Figure 2D). A stick diagram represents the different hindlimb positions. Normalizing these positions to the coordinates of a reference point (the tail base) eliminates minor variations unrelated to limb movement, facilitating a more accurate understanding of the motion (Figure 2D). In this particular mouse, ankle extension rapidly increases in the middle of the movement. This is further supported by the plot of angle variations over time (Figure 2E). This shows that the slope of the angle opening is steeper between 125 and 200 ms, while it is less pronounced at the start and end of the movement.

The next example involves an adult mouse linear swimming test, recorded from the side to observe the movement of the hindlimb. The stick diagram, illustrates the position of the hindlimb throughout the movement (Figure 2F). Although the *Stride & Gait module* of the software was initially developed for analyzing gait cycles, it can be also used to evaluate other types of locomotion, such as swimming. In rodents, the swim cycle can be divided into two phases: the power, and the return, strokes (Gruner and Altman, 1980). The power stroke (depicted in orange) involves a backward extension of the hindlimb from the most flexed to the most extended hip angle position (Figures 2F, G). The return stroke (depicted in gray) involves the hindlimb moving forward at a slower pace until reaching maximal hip flexion, while the mouse glides in the water. To analyze this cycle, we used the maximal hindlimb extension following the power stroke as the entry position. When applying these criteria to three swim cycles, we found the power stroke duration to be 35, 40, and 25 ms, while the return stroke duration was significantly longer, measuring 100 and 110 ms (Figure 2G).

**FIGURE 3:**
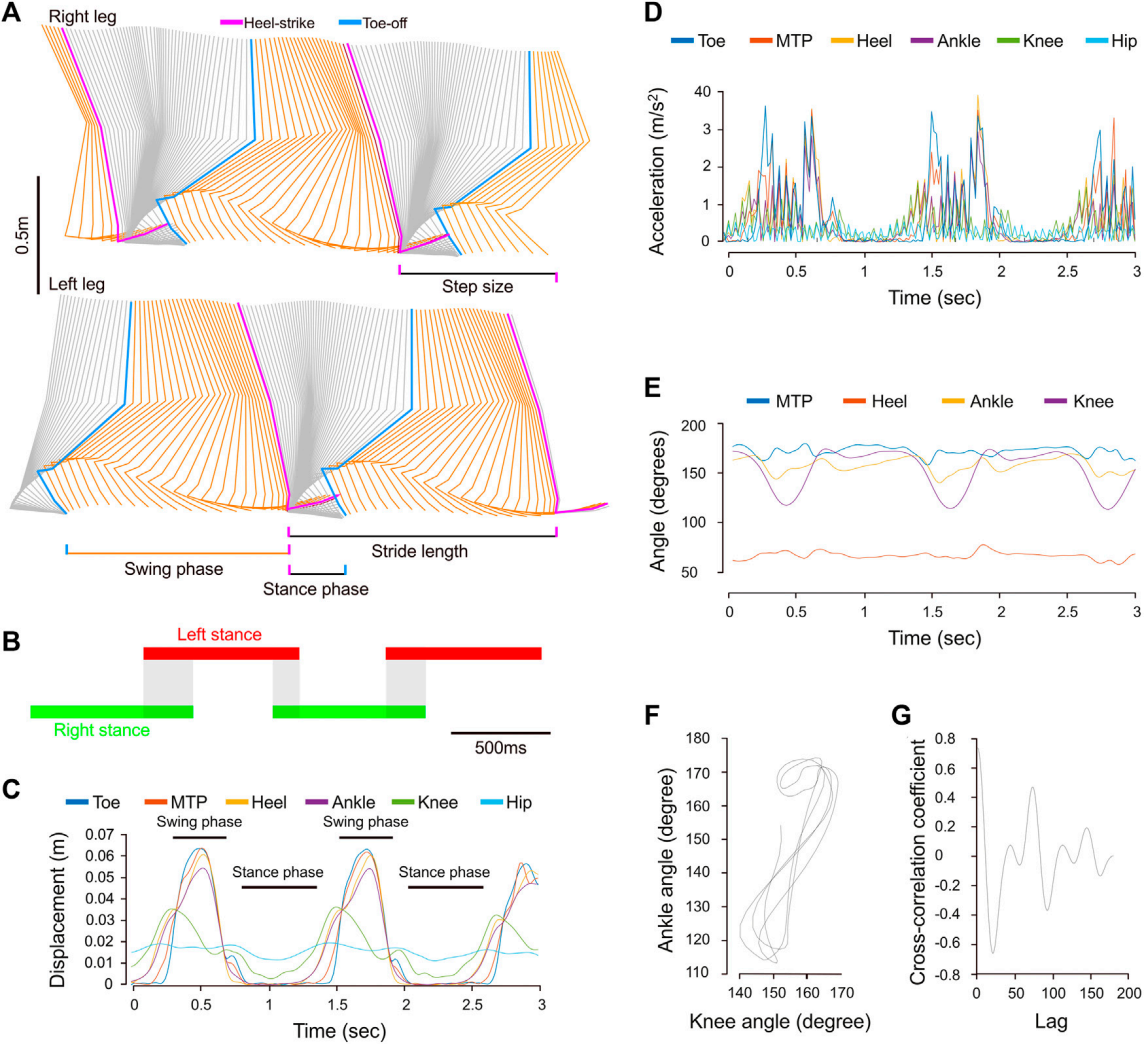
Example showcasing the utilization of MotionAnalyser for analyzing human gait. **(A)** Stick diagram depicting the sequential positions of the hindlimb of the right and left legs. The swing phase is depicted in orange, while the stance phase is represented in gray. **(B)** The gait diagram illustrating the duration of left (red) and right (green) stance phases, as well as the overlap between them (gray). **(C)** Temporal displacement curves for various anatomical points of the right leg. Peaks in displacement indicate the swing phase, while a halt in movement signifies the stance phase. **(D)** Temporal variation of the xy acceleration for the same anatomical points as in **(C)**. **(E)** Temporal variation of the MTP, heel, ankle, and knee joint angles. **(F)** Cyclogram illustrating the relationship between the ankle and knee joint angles throughout the gait cycle. **(G)** Cross-correlogram displaying the relationship between ankle and knee joint angles throughout the gait cycle.

The fourth example shows the capability of MotionAnalyser’s to analyze human locomotion. Walking primarily involves a horizontal displacement of the legs, characterized by cycles of joint flexion and extension, as well as alternation between the left and right legs. This can be easily modeled from coordinates in the x-y-(z) plane using MotionAnalyser to generate stick diagrams and animations (Figure 3A; Supplementary Video S1). In addition, quantitative analysis of the gait cycle can also be obtained. In this example, the gait was characterized by a stance phase of almost 800 ms for a swing phase of 450 ms. The stride duration was 1.2 s, the stride length was 1.15 m, the step duration was 584 ms, and the step size was 0.66 m. The gait diagram also shows an overlap of the left and right stance phases typical for bipedal walking (Figure 3B). The profile of instant displacement, however, is not identical for all leg joints (Figure 3C). While the hip curve presents low variability, the other joints alternate peaks with almost an arrest of displacement. This corresponds to the cyclic occurrence of the swing and stance phases. The joint angle variations are consistent with the displacement. There is also a complex phase relationship between the variation of the ankle and the knee joints (Figures 3E–G). The cross-correlation coefficient of 0.8 at Lag 0 indicates a strong in-phase correlation between these angles, which aligns with the calculated phase shift of 15.5°.

**FIGURE 4:**
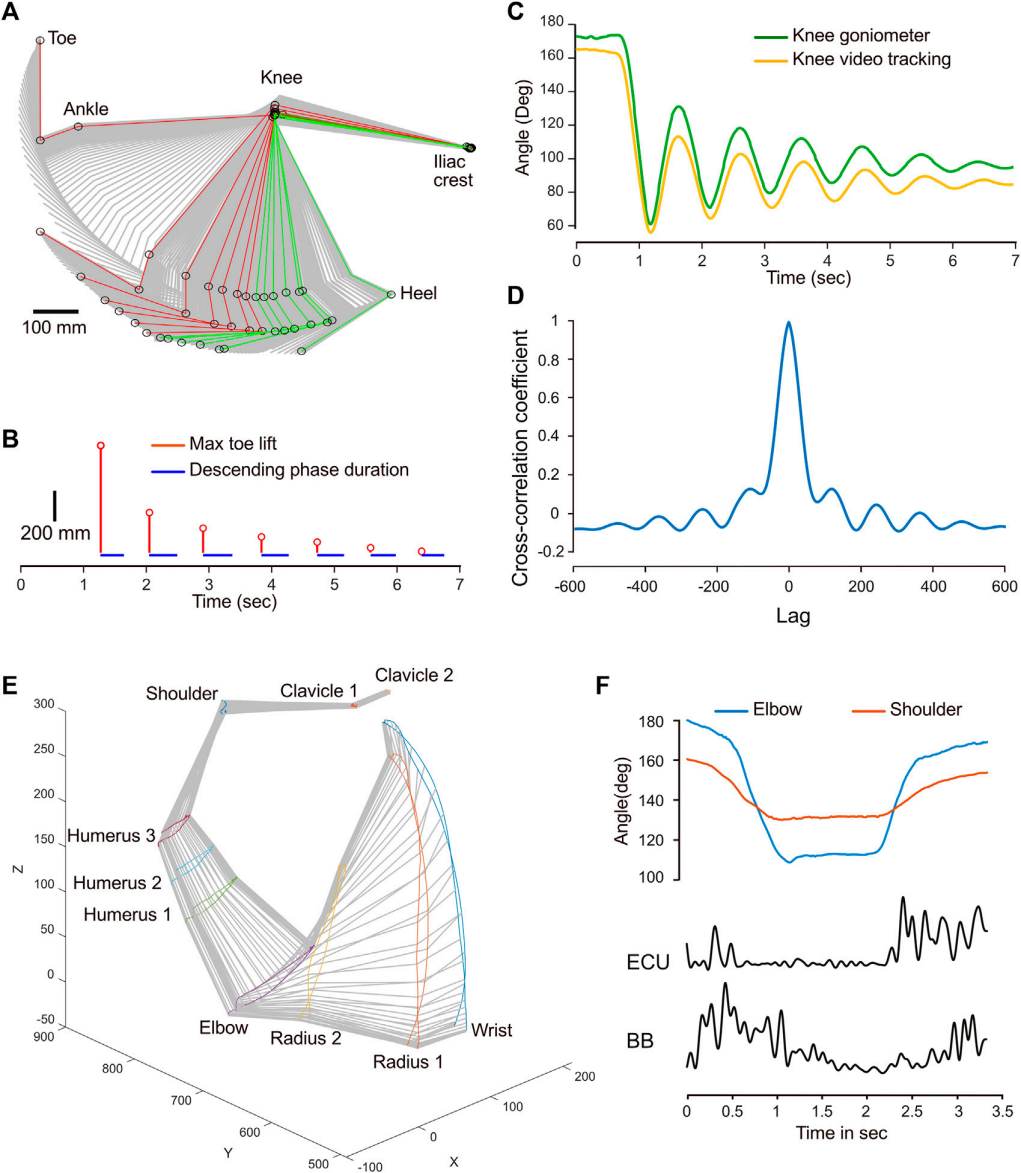
Examples of combined kinematic and EMG or goniometer signals. **(A)** Stick diagram illustrating the sequential positions of the hindlimb during a pendulum test. The positions corresponding to left maxima are depicted in red, while those corresponding to the right maxima are shown in green. **(B)** Temporal stem plot (red) showing the toe lift amplitude decay during the pendulum motion. The blue segments represent the leg’s falling phase duration. **(C)** Temporal angular variation of the knee joint obtained from video tracking (yellow) and a goniometer (green). **(D)** Cross-correlogram depicting the correlation between the knee joint angle variations obtained from video tracking and the goniometer. **(E)** Perspective stick diagram depicting the sequential positions of the arm and shoulder over time during a flexion-extension movement. **(F)** Comparison of the temporal angle variation for the elbow and shoulder joints with the EMG envelopes of the extensor carpi ulnaris (ECU) and biceps brachii (BB) muscles.

For the next example, a leg pendulum test was conducted and the leg joints were tracked, while a wireless goniometer recorded variations in the foot-knee-hip angle. The stick diagram illustrates a typical pendulum movement with a decay shape (Figure 4A). By using the *Stride & Gait* component of the program, the positions that correspond to the left (red) and right (green) maxima were isolated for each cycle. This allowed for the calculation of the cycle duration, which was relatively constant at 460 ms, except for the initial drop, which was faster, with 380 ms (Figure 4B). In addition, the vertical amplitude of the toe for each cycle also agreed with a typical decay process. The reliability of the goniometer—which was positioned to record variations in the knee angle—was tested against the calculated outcome obtained from video tracking. The shapes of the two curves are similar, although noticeable differences were found (Figure 4C). There were 8 degrees of disparity between baselines. Furthermore, the amplitude of the goniogram after the first minima was larger than that from the video tracking data. The cross-correlation coefficient at lag 0 was 0.99, indicating a strong similarity and signals in phase (Figure 4D). However, the correlation becomes weaker and oscillates as the lag increases, reflecting variations in the magnitude of correlation between the signals at different lags and a slight phase drift, which is already visible at 3 s (Figure 4C). These differences are likely to reflect the different modalities used to acquire the data. Tracking from video recording only accounts for a 2D movement, while the goniometer integrates the joint’s angular velocity, introducing drift.

The final example demonstrates a straightforward flexion-extension movement of the arm of a healthy human subject. The coordinates from the motion capture system were imported in MotionAnalyser to create a 3D model of this movement (Figure 4E). The flexion-extension movement is primarily caused by a biphasic angle variation in the elbow and shoulder joints (Figure 4F). Processing EMG signals with MotionAnalyser for the biceps brachii and the extensor carpi ulnaris also reveals a biphasic curve, with muscle contractions occurring during the dynamic phases of flexion and extension.

Altogether, these results show that MotionAnalyser is a reliable and versatile tool that enables an integrative analysis, where motion can be confronted to electrophysiology and to sensor signals for a better understanding of the biomechanics of the movement.

## Discussion

All the free and open source software packages described in the introduction implement several kinds of tracking algorithms suited for a plethora of different experimental setups and conditions. However, they mostly compute basic kinematic quantities. Derivation of higher order kinematics and its subsequent analysis is left to the researcher. Our software, instead, places itself in a somewhat opposite direction: given the position of the points of interest (which requires the tracking problem to be already solved), it computes basic and higher order kinematic variables and produces multiple plots commonly used in the analysis of motion behavior, thereby streamlining the data analysis process. Other software packages like OpenSim (Seth et al., 2018) are suitable for advanced kinematic calculations and for simulation of movements based on a skeleton model. In general, the software packages for combinatorial analysis of movement and physiological data require programming skills for installation and use. In contrast, our software aims to provide a user-friendly analysis platform that does not require coding skills. Although we found some overlap between the functions of MotionAnalyser and those of Kinovea, the latter specializes in humans and sport science, offering little generalization for animal models, and does not support 3D analysis from motion capture and gait analysis. Neither does it support the processing of electrophysiology traces and sensor signals for an integrative movement analysis. The proprietary solutions offer functionalities analogous to MotionAnalyser. Some of them are superior, for example—they integrate 3D motion capture with real-time animation and skeleton overlay, with or without muscles. They also propose different solutions to combine motion capture with external recording devices, such as force plate and EMG recording. However, they incur significant costs, do not always offer trial versions, the underlying code is often proprietary, and, in some cases are bound to a proprietary acquisition system, which further increases costs and may lack adaptability and decrease reusability of the equipment already acquired.

Using artificial datasets, we demonstrated that MotionAnalyser is a reliable tool. In addition to data analysis, the software offers a range of features that enhance user experience. For instance, importing data from different kinds of equipment for a multimodal analysis can be challenging, especially for users with limited computer skills. MotionAnalyser has a streamlined process for import and export of data in various file formats. In addition, the software handles datasets separately, enabling users to analyze them independently while displaying them together (if desired). This feature is particularly valuable for a decomposition of limb movement, such as the gait cycle. It is also a convenient tool to simultaneously animate multiple limbs. In addition, the software proposes an extensive toolbox for data preprocessing, such as concatenation and trimming of dataset, scaling, aligning, rotating, deletion of data columns, deletion of incorrect data points, interpolation of missing points, etc. In addition, the program can recalculate the time column and resample or interpolate datasets to a desired frequency, providing a solution to a common obstacle when analyzing data from different recording devices with varying sampling rates. MotionAnalyser also proposes a set of tools for digital filtering, rectifying, smoothing, and calculating FFT.

This software can be valuable in many research fields, such as neuroscience, rehabilitation medicine, sports, robotics, and others that address questions related to movement and coordination in various types of experiments. A typical example of use is for tracking animals in a confined environment, which is a very common approach in behavioral science. Originally, we created MotionAnalyser to analyze limb movements in animals, as illustrated by the vestibulospinal and the swimming tests. Using this, it becomes easy to address problems like balance deficits or malfunction of the motor system (Bachmann et al., 2013; Di Bonito et al., 2015; Züchner et al., 2018; Toch et al., 2020). However, the concomitant use of electromyograms provides more insights into the function, as previously shown for the vestibulospinal test (Lambert et al., 2016). A combination of gait analysis with joint kinematic, electromyograms, and correlation analyses can reveal different relationships between specific parts of the movement. This approach is widely used across different disciplines like basic science (Capogrosso et al., 2016) or physical therapy (Martinez et al., 2022). Furthermore, to address a specific situation, it might be necessary to correlate movement with data from sensor systems, such as inertial measurement units in combination with a force plate (Drobnič et al., 2023). We, therefore, implemented the possibility to import and analyze data from sensor systems. The possibility to work on these different input systems within the same software allows for seamless observation of the different parameters by simply switching between the different tabs. In addition, MotionAnalyser offers a variety of mathematical correlation models that the user can choose from to compare the different kinematic parameters with electrophysiology and sensor system signals. Therefore, MotionAnalyser is a versatile tool that opens up the possibility of multimodal integrative analysis to improve the understanding of function across multiple disciplines.

In biological science and medical research, it is common to create in-house programs that are designed to be used by researchers with certain levels of computer literacy, or even by programmers themselves. In the field of movement tracking, a notable example is the development of DeepLabCut (Nath et al., 2019), a Python-based deep learning program used for tracking animals or limbs even in the absence of markers. Despite the efforts made by the DeepLabCut team to increase user-friendliness, such as providing learning materials through video tutorials and forums, it still requires time and effort for someone without substantial computer skills to establish this tracking platform. The need for more accessible software that does not require user programming skills is highlighted by recent efforts for creating an accessible open-source video analysis pipeline (Pennington et al., 2019). Because MotionAnalyser performs simpler tasks compared to machine learning-based programs, it was more straightforward to design an intuitive and user-friendly interface that necessitates only a minimal number of user-defined parameters. While it would have been simpler and quicker to code MotionAnalyser as a command line program, it was essential to develop a tool that could cater to both advanced users and to those who lack coding skills and the time to develop them. Consequently, we developed a graphical user interface (GUI), enabling users to perform analyses with minimal effort. As of the current version, the software only requires a basic level of computer literacy. There is a growing trend in the development of analysis software utilizing Python (Srinath, 2017). Python is freely available and open source, making it accessible to researchers without any associated costs. Furthermore, Python provides a wide array of libraries with diverse tools and functionalities, facilitating rapid development. However, the Python ecosystem can be quite complex to use, as it requires environment setup, library imports, installation hurdles, and other tweaks. Therefore, our choice fell on MATLAB, which—while carrying the drawback of being closed source and necessitating a license payment—has the advantage of having a straightforward installation process, while the licensing requirements do not apply to end users, thanks to its freely available runtime environment. To encourage other researchers to adopt the tool, we created a short user manual, which constitutes the base for a wiki that we are building. Additional resources include video tutorials and various example datasets. All resources are freely available for download at https://github.com/BoullandLab/MotionAnalyser. To increase the dissemination of the program, the software is released both as a .mlapp package and as a standalone installer for both Windows and Linux. Source code is available under the terms of GPLv2.

MotionAnalyser, however, has limitations that can be addressed in different ways; we will provide support for new functions and bug fixes for future releases of the program. At the moment, users deeply interested in gait analyses may find that the program is missing the option to calculate torque. Torque in human gait can be calculated using various methods, from EMG signals or by using force plates to capture the forces and moments exerted on the foot during walking or running. Given MotionAnalyser’s current ability to handle EMG and sensor signals, adding torque calculation is possibly one of the first improvements that have to be considered. Another interesting improvement could be to implement calcium imaging, which could be particularly interesting to correlate with motion. The only possible way to do that at the moment is to import values from regions of interest in the *E-Phys* tab. Being able to handle the image analysis directly from MotionAnalyser could be an interesting improvement. It has also been proposed to implement the analysis of audio tracks, which could be relevant in different experimental setups like the assessment of echolocation during flight maneuvers in bats (Falk et al., 2015). Yet, at the moment, the only possibility is to import the audio trace as an electrophysiogy signal. This gives access to the filtering provided in the *E-Phys* tab, though it may not be extensive enough for this purpose. Another improvement for a future release could be to enhance the animation tab with the possibility to fit the tracked POI to a skeletal model. A technical improvement to increase the exporting speed of video or animated gif files could also be interesting, since benchmark tests have revealed this process to be the slowest in the program. Further development of preprocessing tools could also be interesting. For instance, in the present version of the software, the dataset stitching feature primarily allows users to align and concatenate datasets based on their visual overlap. However, it does not offer camera calibration options and adjustments to compensate for distortions and smoothly blend the coordinates. Implementing these transformations in a future release of MotionAnalyser could also be considered. We encourage the use of the issue tracker of the repository to request additional features and report bugs.

Despite these limitations, MotionAnalyser is an appealing software because of its accuracy, user-friendliness, versatility, free access, and open-source code. It is designed to cater to a wide range of researchers across various disciplines, regardless of their level of computer literacy.

## Data Availability

The analysis software described in this article is free and open source. It is available for download, along with example data files, from https://github.com/BoullandLab/MotionAnalyser.

## Supplementary material

SUPPLEMENTARY FIGURE S1 Examples of data preprocessing functions for adjusting a coordinate dataset. **(A)** Digital filtering (30 and 35 Hz) of a dummy sine signal containing components at 25, 30, and 35 Hz. **(B)** Fast Fourier Transform of the signal before and after sequential application of the 30 and 35 Hz Notch filters. **(C)** Importing coordinates for the joints of the left and right leg as a single set creates a model of the leg joints attached by a segment. Importing the left and right leg coordinates independently results in a correct representation. **(D)** Correction of the mirror effect by inverting x-coordinates. **(E)** Translation of x and/or y-coordinates to align legs on a desired point. **(F)** Rescaling of the dataset. **(G)** Trimming the dataset. **(H)** Reduction of segmentation density for displaying high-frequency data points.

SUPPLEMENTARY FIGURE S2 Sequence of actions required to stitch coordinates. **(A)** Dataset from Camera 1 shown in grey **(B)** Positioning of the dataset from Camera 2 shown in orange. **(B)** Determination of the stitching position (green position). **(D)** Concatenation of the two datasets.

SUPPLEMENTARY FIGURE S3 Examples of signal treatment options offered by MotionAnalyser. **(A)** Fast Fourier Transform of an EMG signal. **(B)** Spectrogram for the same EMG signal. **(C)** Raw EMG signal used in **(A,B)**. **(D)** Digital filtering and rectification of the signal. **(E)** Signal envelope.

SUPPLEMENTARY FIGURE S4 Benchmarking of different functions of MotionAnalyser using a mid-range computer and five different input files containing an increasing number of datapoints from 1,300 to 13 million values. **(A)** Time for importation of raw values. **(B)** Time for plotting a stick diagram. **(C)** Time for saving/exporting data after analysis using different file formats. **(D)** Time for various functions.

SUPPLEMENTARY FIGURE S5 Accuracy of MotionAnalyser for biomechanics analysis. **(A)** Dummy array of coordinates with an affine x-component and a quadratic y-component. **(B)** Temporal variation of x- and y-speed and acceleration calculated from the dummy arrays shown in **(A)**. **(C)** Dummy array for creating segments attached at a known angle. A, known angle; MA, angle calculated by MotionAnalyser. **(D)** Dummy array for emulating human gait, depicting the different leg positions: heel strike (red), heel-off (green), toe-off (blue), maximum toe-lift (magenta), and intermediate positions (gray). Data are expressed as arbitrary units (Au). **(E)** Gait diagram generated from the dummy array **(D)**. **(F)** Position of a mouse hindlimb at a specific time point during linear swimming, overlaid with the lines from three protractors centered on the hip, knee, and ankle joint. The readout from the protractor (cyan) is identical to the values calculated by MotionAnalyser (green). **(G)** Comparison of values calculated by MotionAnalyser using a real dataset with values calculated by ImageJ, spreadsheet calculation, or obtained by manual measurements. For better visual comparison, the velocity and acceleration values were divided by 100 and 10,000 respectively, while the gait parameters were multiplied by 10.

SUPPLEMENTARY FIGURE S6 Accuracy of MotionAnalyser for signal treatment and correlations. **(A)** Digital filtering (30 and 35 Hz) of a dummy sine signal containing components at 25, 30, and 35 Hz. **(B)** Fast Fourier Transform of the signal before and after sequential application of the 30 and 35 Hz Notch filters. **(C)** Dummy sine signals of known amplitude ratio and phase shift for testing correlation functions. **(D)** Auto- and cross-correlation from the signals in **(C)**. Note that the auto-correlation of f_2_(x) overlaps that of f_3_(x). For visualization purposes, f_2_(x) has been slightly shifted on the right.

SUPPLEMENTARY TABLE S1 Details of the key functions used in coding MotionAnalyser, including mathematical formulas and MATLAB code.

SUPPLEMENTARY VIDEO S1 Animated model of human walk.
