# Supplementary figures and images for: Integrated software for multi-dimensional analysis of motion using tracking, electrophysiology, and sensor signals
Source: Front Bioeng Biotechnol. 2023 Nov 22;11:1250102. doi: 10.3389/fbioe.2023.1250102 (PMC10703477; doi:10.3389/fbioe.2023.1250102)

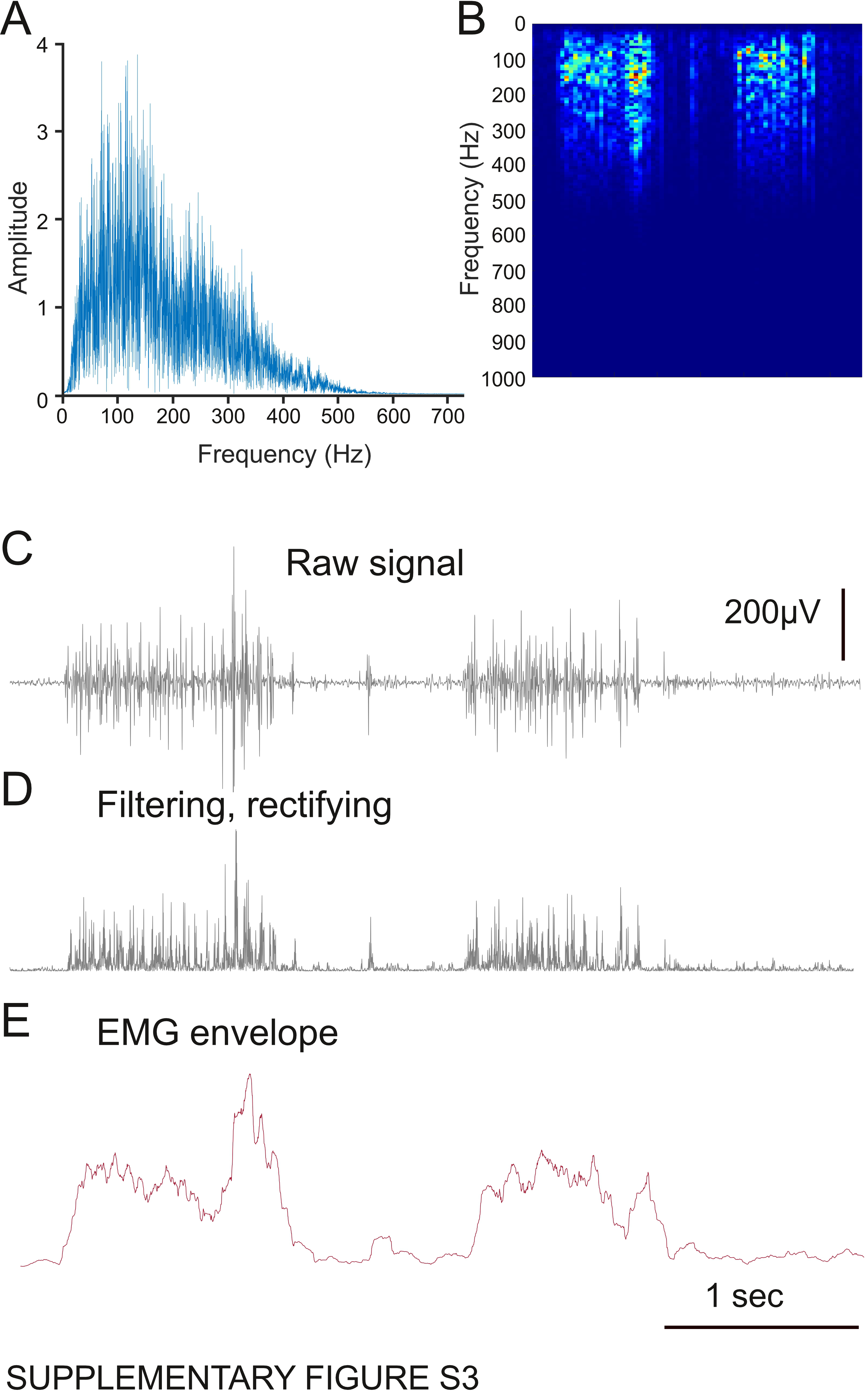

Supplement: Supplementary file 1 [file Image3.jpeg]

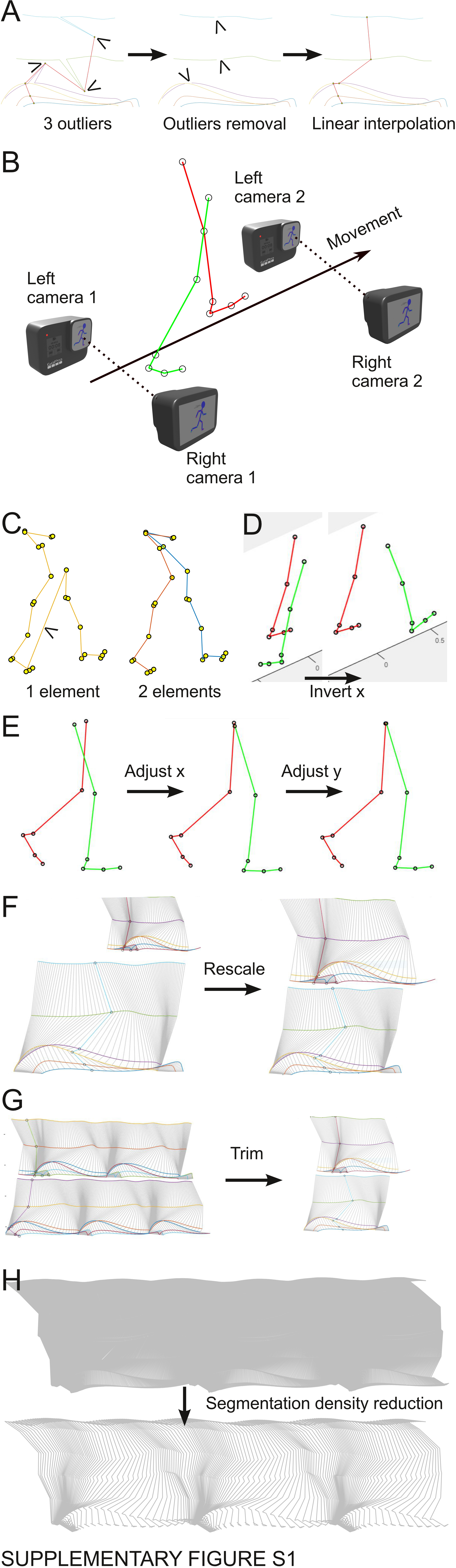

Supplement: Supplementary file 2 [file Image1.jpeg]

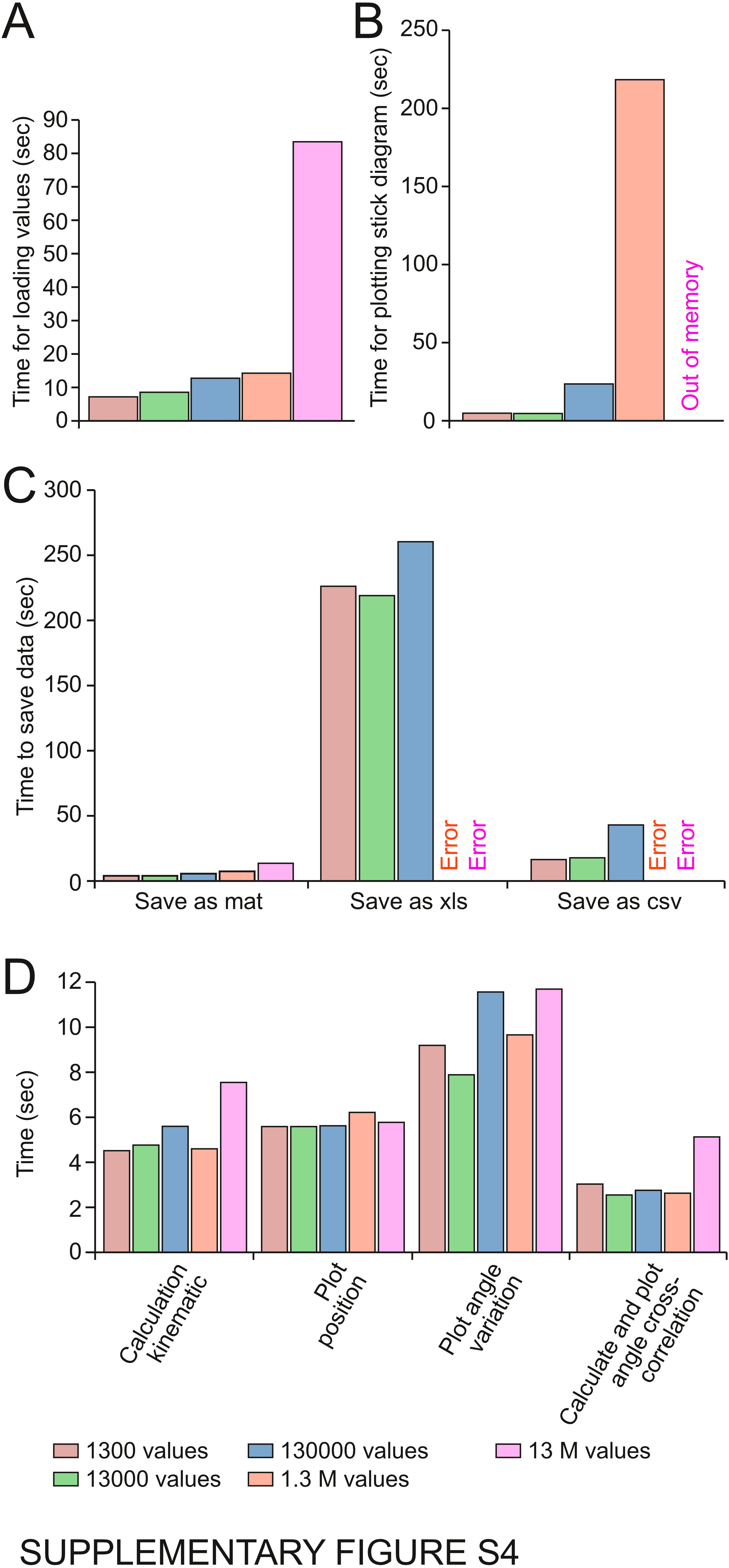

Supplement: Supplementary file 3 [file Image4.jpeg]

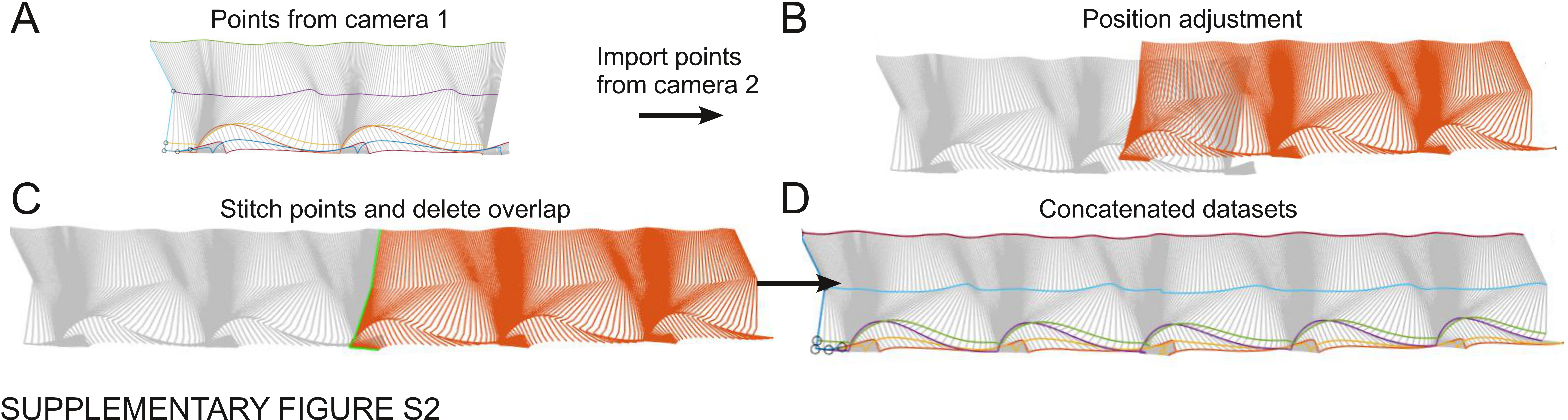

Supplement: Supplementary file 4 [file Image2.jpeg]

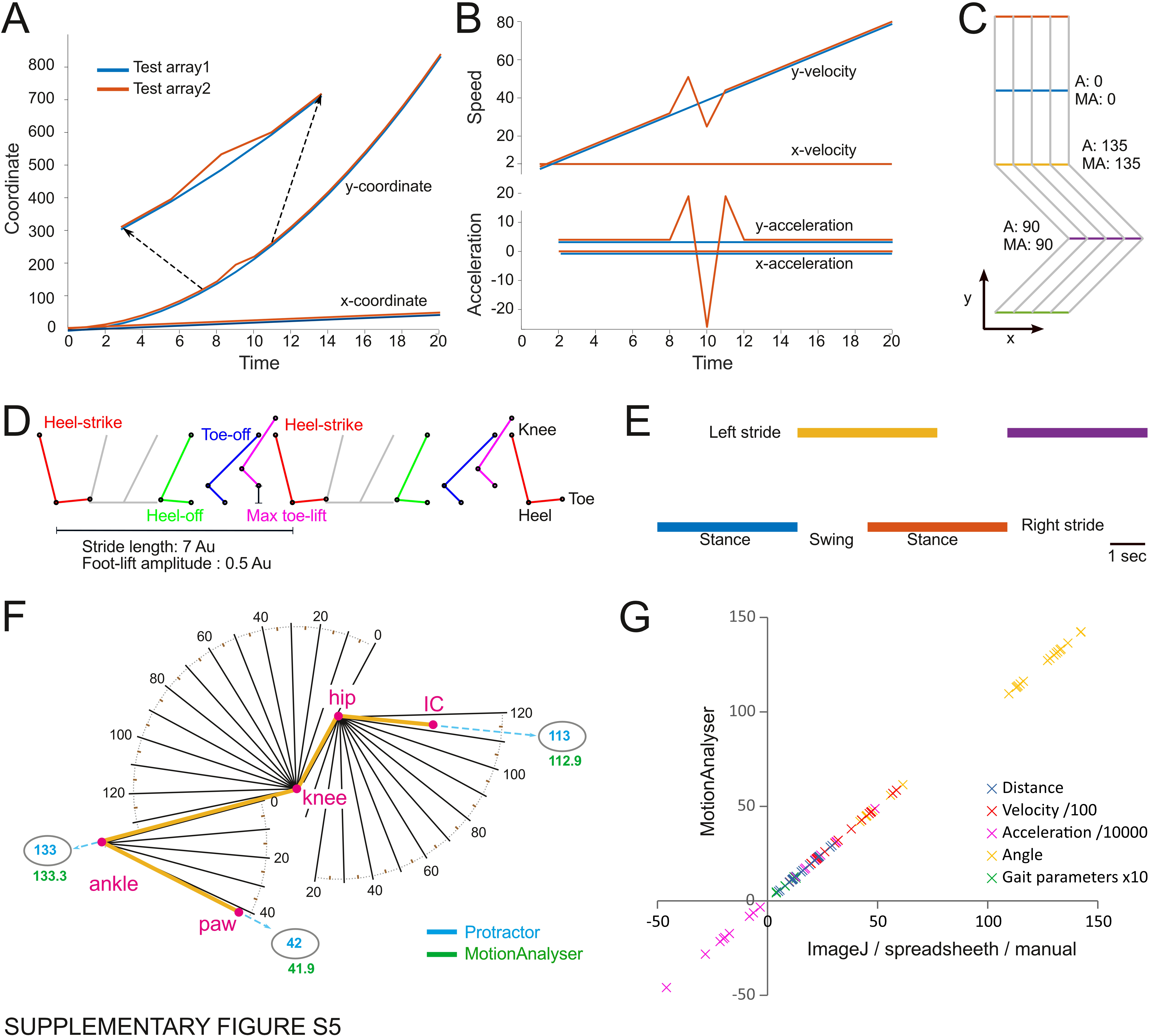

Supplement: Supplementary file 5 [file Image5.jpeg]

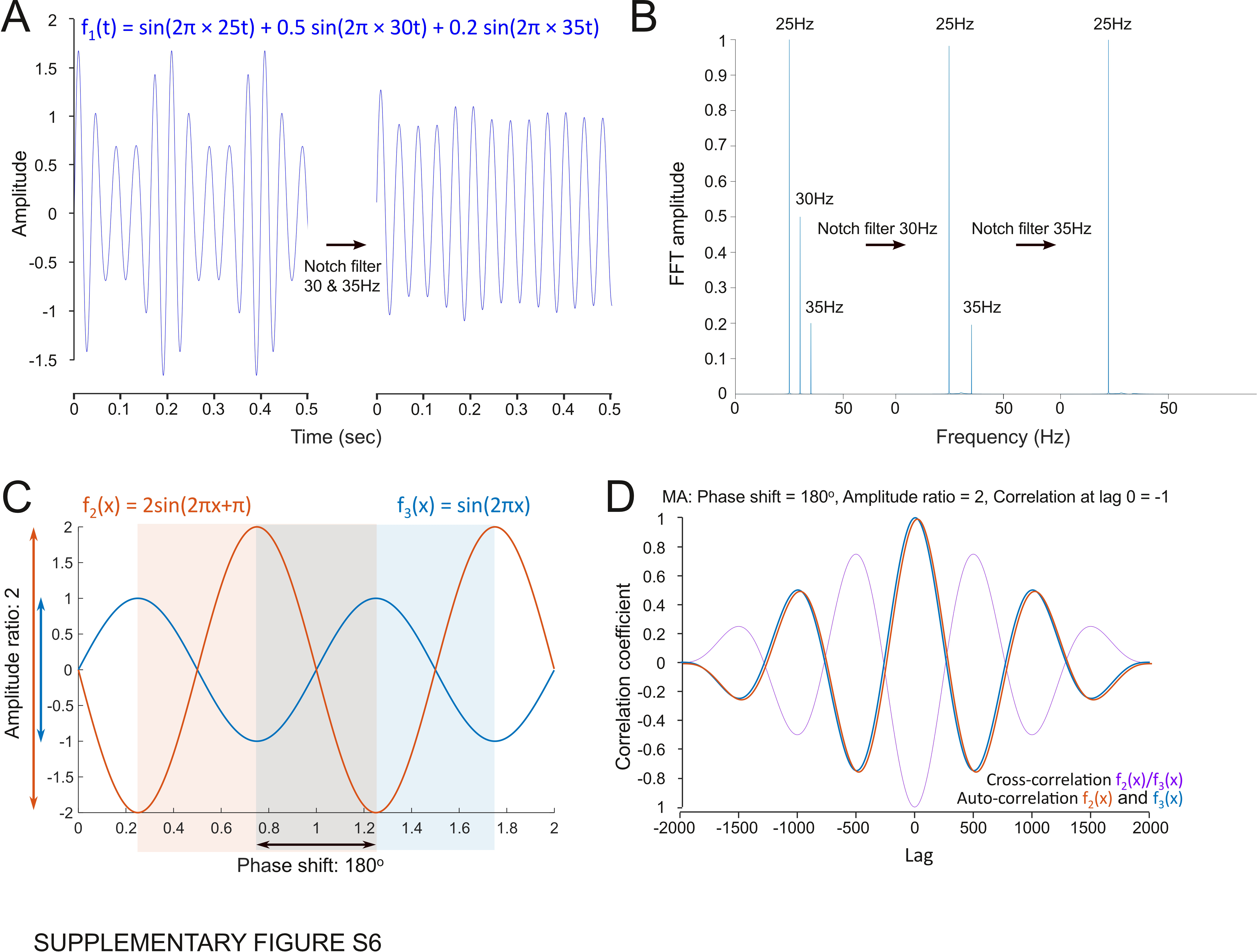

Supplement: Supplementary file 8 [file Image6.jpeg]
